# Cuts and the cutting edge: British science funding and the making of animal biotechnology in 1980s Edinburgh

**DOI:** 10.1017/S0007087417000826

**Published:** 2017-12

**Authors:** DMITRIY MYELNIKOV

**Affiliations:** * Centre for the History of Science, Technology and Medicine, University of Manchester, Simon Building, Manchester M13 9PL, UK. Email: dmitriy.myelnikov@manchester.ac.uk.

## Abstract

The Animal Breeding Research Organisation in Edinburgh (ABRO, founded in 1945) was a direct ancestor of the Roslin Institute, celebrated for the cloning of Dolly the sheep. After a period of sustained growth as an institute of the Agricultural Research Council (ARC), ABRO was to lose most of its funding in 1981. This decision has been absorbed into the narrative of the Thatcherite attack on science, but in this article I show that the choice to restructure ABRO pre-dated major government cuts to agricultural research, and stemmed from the ARC's wish to prioritize biotechnology in its portfolio. ABRO's management embraced this wish and campaigned against the cuts based on a promise of biotechnological innovation, shifting its focus from farm animal genetics to the production of recombinant pharmaceuticals in sheep milk. By tracing interaction between government policies, research council agendas and local strategies, I show how novel research programmes such as genetic modification could act as a lifeline for struggling institutions.

## Abbreviations

ABRO: Animal Breeding Research Organisation (Edinburgh, 1946–1986)
ABRC: Advisory Board for the Research Councils
ACARD: Advisory Council for Applied Research and Development
AFRC: Agriculture and Food Research Council (1983–1994, formerly ARC)
ARC: Agricultural Research Council (1931–1983)
BBSRC: Biotechnology and Biological Science Research Council (replaced AFRC in 1993)
IAPGR: Institute of Animal Physiology and Genetics Research (Edinburgh and Cambridge, 1986–1993)
JCO: Joint Consultative Organisation for Research and Development in Agriculture and Food
MAFF: Ministry of Agriculture, Fisheries and Food
MRC: Medical Research Council
PRC: Poultry Research Centre (Roslin)

On 21 December 1981, Gavin Strang, the Labour MP for Edinburgh East, launched a passionate speech in the House of Commons. Its subject was the proposed 80 per cent cut to the Animal Breeding Research Organisation (ABRO), an institute where he happened to have worked before starting his Parliamentary career. Strang defended the unique place of the organization in British research, its high scientific standards and its great contributions to meat production and breeding efficiency. Responding, William Shelton, a junior science minister in Margaret Thatcher's first government, argued that the proposed cut had been fully the decision of ABRO's funder, the Agricultural Research Council (ARC) – now the Biotechnology and Biological Sciences Research Council (BBSRC).^1^ Shelton claimed that the council sought to reduce ABRO to a small group of high-flying scientists, move breeding research to university laboratories, and redirect resources into high-priority areas, such as biotechnology. In the following years ABRO avoided the 80 per cent cut, but it did lose about half its funding in 1982. Fewer than fifteen years later, Dolly the sheep was cloned at ABRO's direct descendant, the Roslin Institute, to global acclaim and infamy.^2^ How, in that time, did an institute on the verge of closure come to achieve such an iconic feat?

This article examines ABRO and its successors at a time of crisis. I trace the changes in science policy as they were felt, interpreted, implemented and resisted. The transformations at ABRO have been absorbed into the narrative of Thatcher's general assault on public institutions, and indeed top-down changes to funding and accounting mechanisms were an important causal component.^3^ A specific examination of the events, however, suggests a more complex picture, and shows strong continuities with the policies of the 1970s. By paying attention to the interplay between macro and micro levels, this paper explores how governance can change research direction at a given place. Furthermore, while there had long been interactions between the farm and the clinic, the reforms at ABRO happened during the transition to the biotechnological mode of doing agricultural science, where consumers and innovative companies rather than farmers became key stakeholders.^4^

The figure of Margaret Thatcher is symbolic of the dramatic transformation of British life in the 1980s, and it is tempting to see changes at ABRO as an application of Thatcherite policies to a research institute.^5^ Yet, while agricultural research suffered greatly during Thatcher's tenure, the ABRO story does not fit this frame comfortably. First, the reorganizations were a consequence of the 1971 customer–contractor policies initiated by Lord Rothschild, head of Edward Heath's Central Policy Review Staff, and implemented in a way that proved unstable a decade later. Second, the decision to heavily curtail ABRO research in 1981 pre-dated serious cuts to the ARC's budget, albeit by months, not years. There was an element of anticipating future austerity, yet the decision was largely driven by the ARC's wish to free funds to invest into new high-priority areas: plant nutrition, animal health and especially biotechnology. Facing this decision, some at ABRO saw the crisis as a way to promote their research agendas and loosen the organization's reliance on breeding experiments, favouring instead reproductive physiology and the introduction of molecular genetics. For the new management at ABRO, the obsession with biotechnology became a lifeline as they decided to appeal to the ARC by promising a significant programme of animal genetic engineering, and delivered impressive results within a decade.

US-focused histories of biotechnology in the 1970s and 1980s tend to emphasize the importance of enterprise, commercialization and private investment in the conduct of research.^6^ The role of US federal government was limited to regulating (and deregulating) amid concerns about safety, and to creating a climate for the new industry, notably through intellectual-property legislation.^7^ Studies of biotechnology in the UK, by contrast, have uncovered an alternative approach from the British state that focused less on intellectual-property protection and more on government support and investment.^8^ The story of ABRO and its successors offers a hybrid of these accounts. Government funding – and its withdrawal – were crucial in setting up the biotechnology programme at the organization. At the same time, ABRO sought alternative links and a more stable income in conjunction with industrial partners. ABRO pursued patents and entered an agreement with a private Scottish company, PPL Therapeutics, which licensed technologies in exchange for research support. Pushed both to build industrial links and to pursue more ‘basic’ or ‘fundamental’ research, ABRO used genetic modification to develop a niche that answered both demands.

This article proceeds chronologically. I begin by discussing the major changes to science funding that took place in 1972 as a result of the Rothschild report, which greatly affected the ARC. The next section discusses the ARC's financial position in the early years of the first Thatcher government, and explains its decision to heavily curtail ABRO research. The following section examines ABRO's response to the cuts and its campaign of resistance to the council's decision, which resulted in a 50 per cent settlement and a change of outlook. I then explore the adoption of molecular biology as a promising research agenda. The final section traces the further cuts to the ARC and ABRO's decision to complement its state funding with private support for genetic engineering work.

## ABRO, the ARC, and the Rothschild report

The UK Agricultural Research Council (ARC) was set up by Royal Charter in 1931, incorporating a number of pre-existing agricultural research institutions. Its mission was to centralize agricultural research driven by ‘pure’ science, with funding decisions made by scientists. Modelled on the Medical Research Council, the ARC enjoyed extensive autonomy and received its funds directly from the Department of Education and Science, in an annual allocation commonly known as the ‘science vote’. The arrangement followed a battle with the Ministry of Agriculture and Fisheries, which had campaigned for research closely aligned with governmental agricultural policy, under ministerial supervision.^9^ The 1956 Agriculture Research Act cemented the ARC's autonomy from the ministry in choosing which research it funded. Despite its mission of pursuing pure science that could then be extended to agricultural practice, the ARC also funded what was recognized as a healthy applied-science programme.^10^ Still, the council remained only a part of the byzantine agricultural research structure in Britain. Private R & D aside, the Ministry of Agriculture, Fisheries and Food, as it became in 1955, had its own, more problem-oriented, bodies around the country with a focus on extending scientific advances to farmers, known jointly as the National Agricultural Advisory Service.^11^ Furthermore, the Department of Agriculture and Fisheries for Scotland enjoyed a certain autonomy and had yet another network of research sites.

**Figure 1.**
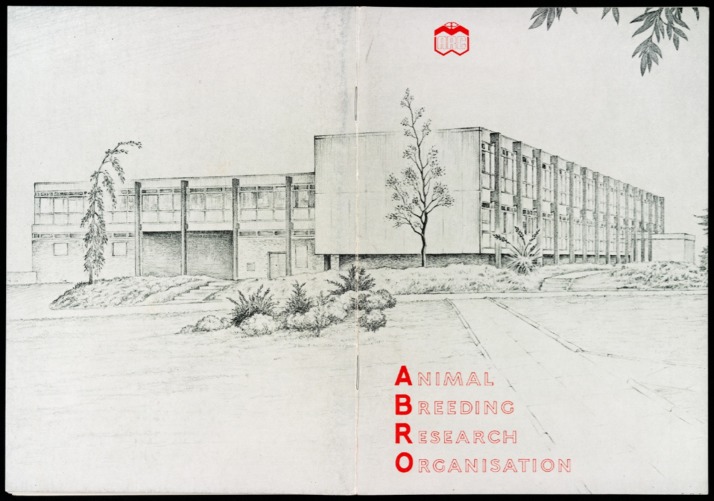
Brochure for the Animal Breeding Research Organisation showing its new headquarters at the King's Buildings site in Edinburgh, n.d., *c.*1970. Reports relating to the Agricultural Research Council (5.07), 1951–1983, Roslin Papers, Edinburgh University Main Library, EUA IN23/1/1/1. Courtesy of Edinburgh University Main Library, distributed under a CC-BY license.

Despite Scottish autonomy in funding agricultural research, the ARC set up its own institutions there, managed from London. In 1945, the National Animal Breeding and Genetic Research Organisation was established in Edinburgh as a research institution and an advisory body for farmers, and was renamed the Animal Breeding Research Organisation in 1951. The aim was to build on the animal breeding expertise already present in the city, mostly concentrated within the Institute of Animal Genetics set up by James Ewart and F.A.E. Crew after the First World War.^12^ ABRO soon started a considerable research programme, joining the ARC's portfolio of research institutes, each focusing on a specific agricultural area. ABRO's mission was to bring the knowledge of animal genetics to farmers, assess breeding techniques and conduct its own crosses on experimental farms, working with pigs, sheep and cattle. While not part of the University of Edinburgh, the organization maintained connections with geneticists there, aided by its 1964 move to a new headquarters at the King's Buildings, the university's science campus in the city's southern suburbs. Like other ARC institutes, ABRO received a block grant that enabled planning and infrastructure for long-term experiments following farm animals through many generations of breeding and experimentation (Figure 1).

As funding for science grew in the 1950s and 1960s, the scope of work at ABRO expanded. While most focused on quantitative genetics, the institute also attracted expertise in immunology, wool research, animal physiology and pathology, adding laboratories to complement the mostly desk-based quantitative research. By 1970, it had become a healthy research body comprising nineteen senior scientists and running seven experimental farms: five in Scotland, one in England and one in Wales. Some projects were small and resembled university research on a short grant cycle. At the same time, large-scale and ambitious experiments were being initiated in the 1970s: the Hereford project, set to examine selection and physiological genetics of this popular cattle breed; and the multibreed project investigating genetic variation within and between cattle breeds, exploring the benefits and costs of interbreeding.^13^ During the expansion, a cultural division emerged between the largely office-based ‘breeders’ and the laboratory-oriented ‘geneticists’, and while they coexisted happily in the years of plenty, tensions would emerge at a time of crisis.^14^ Still, ABRO's focus on productivity, combined with building wide-ranging expertise in farm-animal biology, was consistent with the ARC's post-war mission to improve agriculture through science, ensuring that Britain would never need to face rationing again.

The tensions between the ARC and MAFF persisted through the 1960s, as the ministry never gave up its plans to take over control of agricultural research. In 1970, MAFF's frustration resulted in the setting up of the Osmond Committee at the Civil Service Department, to investigate the option of bringing the ARC under ministerial control.^15^ This tension triggered two further reviews that ended up bringing striking change to the landscape of British science policy and funding. The first study, by the Council for Scientific Policy within the Department of Education and Science, concerned the relationship of research councils with the state. It was led by Sir Frederick Dainton, a chemist and the Council for Scientific Policy's chairman. The Dainton inquiry started in 1970, shortly after the Conservative Party under Edward Heath had won the general election, on a platform of liberating British enterprise and cutting inefficiencies in spending. As part of his agenda, Heath sought to transform government machinery towards more rational and evidence-based policy making. Shortly after moving to 10 Downing Street, he introduced a new body to advise the Cabinet, independent of ministries and departments and responding directly to the prime minister. Officially called the Central Policy Review Staff, it was headed by Victor Rothschild, 3rd Baron Rothschild. The body soon became known as ‘the Think Tank’.^16^

As one of its first tasks, the Think Tank was charged with reviewing the way government departments and ministries commissioned and managed research, going beyond the Dainton inquiry.^17^ Both reports were eventually published under the same cover in November 1971 as a Green Paper, a draft policy to be discussed. The White Paper, official government policy based on these reports, appeared in July 1972. Together, they made for a curious read. Dainton's tone was level-headed and mundane; he found the system largely satisfactory, if slow to respond to changing circumstances, and recommended a new body to coordinate between the councils. Rothschild's report, by contrast, was universally recognized as provocative, written in lively, pugnacious prose that *Nature* described as ‘a kind of racy telegraphese’.^18^

Rothschild's crucial argument was that scientists and research council bureaucrats, no matter how well intentioned and knowledgeable, were not the people to decide what kind of research the country needed. Rather than trust scientists to come up with most useful directions, Rothschild left the decision to ministries and departments, the ultimate stakeholders. Applied science was to be funded on a customer–contractor basis, i.e. the government customer would commission specific research, and a research body would carry it out as a contractor. Moreover, Rothschild recommended replacing the Council for Scientific Policy with an umbrella body representing all the research councils and defining long-term strategy, the Advisory Board for the Research Councils (ABRC).

Rothschild's stance offered a sharp and deceptively naive division between ‘pure’ and ‘applied’ research. As historians of science have shown, the boundaries between those categories and the very way of defining them have been historically specific, often controversial, and politically driven.^19^ Many of Rothschild's contemporaries saw sharp distinctions as unhelpful and problematic, while the critics of his report appealed to the unity of science and the unpredictability of useful applications that could come out of research envisioned without practical aims. Rothschild, by contrast, contended strongly that the needs of the country were too ‘urgent and identifiable to rely on chance discoveries – to warrant those needs being left to a form of scientific roulette’.^20^ ‘Pure’ or ‘basic’ research was spared, as Rothschild did not appear to consider it was up for negotiation and left it to the Department of Education and Science to fund. In his proposal, research councils would also contribute to what he called ‘general research’ – that is, long-term work with no clear outcomes but of potential relevance to future practical needs – through a 10 per cent surcharge paid by the relevant customer.

**Figure 2.**
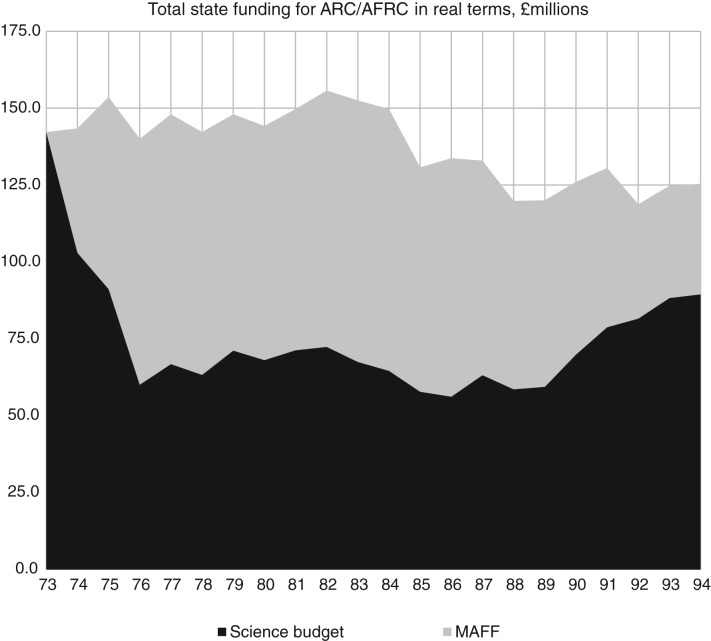
Total state funding for the Agricultural Research Council (Agricultural and Food Research Council since 1983) by year, scaled to 1993 GBP. Based on data in Colin Thirtle, Paolo Palladino and Jenifer Piesse, ‘On the organisation of agricultural research in Great Britain, 1945–1994’, *Research Policy* (1997) 26, pp. 557–576.

Rothschild's role and connections to the prime minister gave his analysis much weight with the Cabinet, and his customer–contractor principle became official policy in 1972. Subsequent governments defended it as sound throughout the 1970s and 1980s. For research councils, the new policy meant that parts of their funding moved from the science budget to a government department that came closest to constituting a customer. Thus the Department of Health and Social Services took over some of the MRC funding, but the MRC managed to reverse the arrangement in the early 1980s.^21^ The ARC, on the other hand, ended up as the key subject of the policy experiment.^22^ About half of the ARC's budget moved to MAFF as its customer – less than Rothschild would have wanted, but dramatic enough (see Figure 2).

The Rothschild report recommended that ABRO should be funded by an ARC block grant from the science vote, without MAFF commissions. This also applied to the Babraham Institute of Animal Physiology and the plant physiology Letcombe Laboratory in Oxfordshire, as all three sites pursued ‘strategic’ research that had no clear applied objectives but was deemed useful.^23^ Strategic research – defined by Rothschild as science designed to develop new applied programmes – become an important category in future discussions about the place of ARC-funded science, especially biotechnology. Yet these recommendations were not adhered to, and the three institutions received mixed ARC/MAFF funding. Some 68 per cent of ABRO's budget moved to MAFF commission.^24^ In the implementation of Rothschild's policy such alterations were common, a result of political expediency and considerable resistance from scientists and scientific bureaucracies, within and beyond the research councils.

In 1974, ABRO's retiring director, H.P. Donald, wrote a piece for the annual report, responding to the recent bureaucratic changes. Titled ‘New tasks for livestock genetics’, it argued that ‘old meaningless phrases about helping farmers and raising efficiency will scarcely serve now’.^25^ Instead, Donald urged ABRO staff to engage more with agricultural policy and industry – not as Rothschild-style contractors, but as consultants who could clarify ill-defined and poorly formulated criteria through scientific theorizing, steeped in genetics. Donald's argument implied that ABRO should embrace theoretical investigations and engage with farming problems.

The ARC did not leave much time for its scientists to act as consultants, but did impose similar expectations in terms of balancing research priorities. ABRO was to pursue both fundamental work of high scientific merit and research with practical applications. The organization was audited every four to six years through the system of visiting groups, composed of senior agricultural scientists, biologists and farmers, who assessed the workings of the institute and the science performed there. The 1974 visiting group at ABRO suggested several changes, notably division of the rigid vertical structure into departments arranged by discipline (see Table 1). Furthermore, the group recommended withdrawing from attempts to produce new breeds, leaving those to the farmers. These recommendations presented the difficult balance the organization had to achieve, as it now had two funders to placate:

That ABRO's principal concerns should be with research directed towards genetic improvement of farm animals … [and with ensuring that] that the Institute [has] competence in relevant basic research; and that while retaining a capability to evaluate the practical validity of the results in its research, the Institute should not put direct effort into developing new varieties of farm animals.^26^

Under Harold Wilson and James Callaghan's troubled Labour governments (1974–1979), Rothschild's ideas continued to be slowly implemented. The Rothschild arrangement splitting pure and applied research appealed to neither MAFF nor the council, and both were eager to avoid sharp cut-offs between the two kinds of science. In order to decide what research might be necessary to contract, the ARC, MAFF and the Department of Agriculture and Fisheries of Scotland set up a Joint Consultative Organisation that started operating in 1973, with five boards dedicated to major agricultural sectors. Each board consisted of between two and six more focused committees, asked to rapidly review the needs in their sectors. In 1975, all boards largely endorsed the status quo, as they argued that British agricultural research had been relevant to farming needs all along.^27^ But the organization also used the opportunity to offer recommendations about promising areas of research. For instance, the Animal Board proposed the following:

Plant breeders are making use of techniques which allow the production of species hybrids and the transfer of relatively specific genetically controlled traits from one species to another. Research on cell and tissue hybrids in animals has for various reasons not reached the same level of advancement and it is recommended that such work should be given some support at the strategic level.^28^

To elaborate on such recommendations, and to make a stronger case for the science vote funding, the Priorities Working Party was established in 1976, comprising ARC and ministry representatives alongside scientists.^29^ In its first report, focusing on plants, the working party recommended several scientific areas of great importance, including nitrogen fixation in soils, studies of photosynthesis and genetic manipulation of crops.^30^ A year after the report was published, Ralph Riley became the new secretary of the ARC (the scientific director balanced by a non-academic chairman). Riley had worked as a molecular biologist at the Plant Breeding Institute in Cambridge, taking over as director in 1972, and had a clear interest in adapting molecular techniques to plant agriculture.^31^

**Table 1.** ABRO's departmental structure, 1974–1986.

Applied Genetics	
Disease Studies	
Growth and Efficiency	
Immunology	
Physiological Genetics	
Statistics and Computing	
Experiments Division (experimental farm management)

Radical changes to science funding presented the ARC with new possibilities. Shared opposition to Rothschild's policy improved the council's relationship with MAFF and allowed the two bodies to articulate new priorities for future research.^32^ MAFF developed its own chief scientist's office and staff, charged with assessing commissioned work, in line with the Joint Consultative Organisation's recommendations. For institutes like ABRO, the benefits were less clear, as MAFF's system of monitoring remained unsettled and threatened interruptions; moreover, the ministry's financial commitments could be withdrawn much more rapidly. Thus, by 1979, MAFF's contributions dropped from 68 per cent to 40 per cent, leaving the ARC to cover the balance. In a 1979 review of the Rothschild framework and its implementation, the Public Accounts Committee criticized the uncertainty and lack of flexibility that research councils now faced. At the same time, it lauded the collaboration between government departments – now ‘customers’ – and the councils, and suggested that greater efficiency would be achieved over time.

## The ARC and Thatcher's first government

Rothschild had left the Think Tank in 1974, but subsequent governments continued to consult him in an informal capacity, especially Margaret Thatcher's. He was not impressed by how the research councils had interpreted his model. In a 1983 letter to Thatcher, he complained,

The scientific community strongly disapproved of my recommendations, and I believe the A.R.C.’s way of impeding their implementation was to set up so cumbersome a bureaucratic machine, ostensibly to implement them, as to make it almost certain that little would happen.^33^

Thatcher's reply was sympathetic. She wrote back, saying agricultural research was in ‘an appalling state of affairs which simply must be dealt with’.^34^ Thatcher had been very familiar with the Rothschild report and its implications – she had been the Education and Science Secretary in Heath's government, although the changes to science policy often happened behind her back. Indeed, in line with her portfolio, she had defended scientific interests and had opposed the customer–contractor principle that ended up becoming official policy.^35^ Much had changed since her time in the Heath government, however. In 1979, Thatcher swept to victory in the general election. As Britain faced dramatic inflation, reaching 18 per cent in 1980, her government embarked on a programme of cuts.

In its early days, however, the Conservative government took it upon itself to protect the science vote. Before its electoral defeat, the Callaghan government had given the ARC a modest increase of £300,000 in 1979 to implement the advice of the Priority Working Party and to hire scientists to work on genetic modification of plants in three of its plant research institutes.^36^ The first year of the Conservative government brought modest cuts that the council could absorb, although it did begin preparing for decreased public spending in the future.^37^ In 1980–1981, there was a further mismatch between funding allocation and growing real-term expenses, to which the ARC responded by refusing to refill a number of vacant posts, and by introducing an early-retirement scheme.^38^ When the expected science vote allocations for 1981–1982 were announced in February 1981, *Nature* celebrated the research councils’ ‘getting off lightly’, with small increases in funding.^39^ Thus the ARC received a £3 million increase to its budget, although much of this was to be spent on growing salary commitments stemming from inflation.

There were crucial changes, however, in how the new government planned public expenditure. Instead of volume purchases – specific spending goals that could be adjusted for salary costs and inflation – the budget was planned in cash, as finite sums.^40^ The ARC's pre-existing commitments were greater than the money available, mostly due to salary increases (committed 7.5 per cent versus budgeted 6 per cent).^41^ As a result, the council had to find more substantial savings. Besides balancing its books, the ARC was also responding to its own wishes, articulated by the Priorities Working Party, to refocus its efforts on more basic and high-profile areas of research, including biotechnology.

Biotechnology was becoming an epitome of high-tech industry where national scientific clout could translate into global leadership and growth. Many historians have argued for the continuity of the ‘new’ biotechnology, based on the set of tools known as recombinant DNA developed in the early 1970s, with older fermenting and chemical industries.^42^ While the revolutionary impact of genetic engineering has been overstated by many, new biotechnology brought dramatic transformations to the life sciences, expanding industrial links, developing patenting practices and establishing scientists’ ability to benefit financially from their discoveries. US universities and start-ups such as Genentech and Cetus led the way, and after the dramatic 1970s debates around the safety of recombinant DNA were neutralized, British scientific and political establishments were keen to get involved.^43^

Crucial in setting out the course for British biotechnology was the Spinks inquiry started by the Labour government in 1978. Headed by Alfred Spinks, the former director of research for Imperial Chemical Industries, it was a collaboration between the Royal Society, the Advisory Council for Applied Research and Development, and the Advisory Board for the Research Councils. The Spinks report noted that biotechnology straddled the divide between basic and applied research, and argued that as ‘strategically applied research’ it was ill-served by the research council system.^44^ It therefore encouraged wide-ranging collaboration, with the Science and Engineering Research Council (SERC) and MRC becoming key sponsors, and a considerable role also envisioned for the ARC. While ideal inter-council cooperation was never quite achieved, biotechnology experienced great expansion in Britain in the early 1980s, with considerable state support.^45^

Combining a budget strain with an imperative to free new funds for biotechnology, the ARC was unwilling to make an across-the-board cut in its expenditure. University grants only made up about 4.5 per cent of the ARC's expenses, and there was a strong incentive to protect those. Instead, it decided to make major savings, couched in the language of ‘flexibility and structural change’, by severely curtailing research at two institutions – the fruit research arm of the Long Ashton Research Station near Bristol, and up to 80 per cent of work at ABRO. The decision was announced in a press release on 11 December 1981. The ARC justified cuts at Long Ashton by arguing that fruit research was already well supported beyond its economic impact.^46^ The reasons for choosing ABRO were more elaborate. The official line was that, while ABRO had delivered much important science in the past, there was little need for long-term large-scale breeding experiments in the future – that they could be managed by smaller groups at universities, while ABRO itself should focus on its strengths, such as reproductive physiology, employing a much reduced staff.^47^

Work at ABRO was far from the greatest area of expenditure for the council, sitting in tenth place out of twenty-two institutions that the ARC supported, but its budget had nevertheless raised some concerns.^48^ In the first months of 1980, before any cuts were mentioned, the ARC had reviewed ABRO's farm portfolio and found it too ambitious and sprawling. The council suggested the sale of farms in Rhydyglafes, Wales, and in Cold Norton near Birmingham. While the review shows some frustration on the ARC's side, it also encouraged ABRO to replace the lost farms with land to be purchased near Edinburgh, hardly an indication of planned shut-down. Many have blamed the 1980 visiting group for the devastating outcome, but the group's report was by no means a condemnation and highlighted much positive work done there – indeed, some of the group's members subsequently expressed surprise that ABRO was singled out for cuts.^49^ It should be noted, however, that even minor criticisms could be damaging, and members may have shared opinions that dissented from the politesse of the written statement. Indeed, as future events suggest, the ARC leadership read the visiting group's report as rather faint praise.

The group that arrived in October 1980 was headed by John Jinks, a senior geneticist at the University of Birmingham. It also included a farmer, two agricultural scientists, another senior geneticist and Anne McLaren, by then an established mammalian developmental biologist with a strong interest in molecular biology.^50^ The group took issue with ABRO's statistics service, finding that it was overwhelmed and the cause of a publication backlog. At the same time, the final report singled out the reproductive physiology department as excellent, and commended the organization's new departmental structure. The overall verdict was:

The Group is generally satisfied with the state of the Organisation and with the competence of the staff. Some areas of excellence have been identified, particularly reproductive physiology, and the Group notes with pleasure the enthusiasm and ability of younger members of staff in several departments.^51^

The group did not go so far as to overturn the council's decision about decommissioning farms in Wales and England, but it did recommend construction of a mouse facility, the purchase of new farms near Edinburgh and the hiring of additional statisticians. These recommendations were supported by a visit from a senior ARC official to ABRO in February 1981, whose correspondence with the council conveyed a less optimistic picture. It emerged during the course of this visit that not all staff were happy with the costly selection experiments then dominating the research agendas: ‘A wider need was identified by some senior staff, to establish a strong core of fundamental work in biology to counterbalance the present heavy emphasis on large scale, long-term selection experiments’.^52^ The statistics problem seemed more dramatic, too, ‘a massive imbalance between the production of data and its handling’ that suggested poor management.^53^

These concerns, combined with financial pressures on the ARC, led to ABRO being singled out. As I discuss below, the ARC faced far greater cuts after 1982, as well as pressures to increase support for food research and university grants. Together, these factors forced the closure, mergers and privatization of many institutes. These future cuts were part of concerted government attempts to reform agricultural research and to move more to industrial players, although the eagerness of established scientists sitting on the research councils to protect universities and have more basic science was also responsible for diverting funds away from agriculture. The cuts at ABRO are understandably often merged with these changes in retrospective accounts. Yet, as I have shown, the decision to curtail ABRO's applied work pre-dated the real crisis at the ARC. Indeed, rather than exemplifying a Thatcherite agenda, this funding trajectory shows strong continuities with science-funding strategies of the 1970s, for instance the case of the Microbiological Research Establishment (MRE) at Porton Down, Wiltshire, a military research facility focused on defence from biological warfare. Faced with closure during the mid-1970s cuts to defence expenditure, MRE was rescued with the help of John Ashworth, chief scientist of CPRS, who built his case by arguing that the establishment's excellent containment facility was perfectly suited to carry out work on recombinant DNA.^54^ This is similar to the strategy that ABRO pursued, albeit in a civilian context – with its expertise in farm animal embryology and the plentiful expertise in molecular genetics at the University of Edinburgh, it sold itself as a perfect place to develop a recombinant DNA programme in livestock.

## ABRO's response to the crisis

John King, ABRO's director, actively campaigned against the cuts by reaching out to farmer unions, breeding societies, science periodicals, Scottish newspapers and a local MP.^55^ *Nature* and the *New Scientist* covered the potential cuts with alarm.^56^ *Farmers Weekly* released a stark editorial and dedicated a spread to celebrating ABRO's unique contribution to British agriculture and to citing alarmed comments from major farming bodies.^57^ For instance, John Thorley, secretary of the National Cattle Breeders’ Association and the National Sheep Association, was quoted saying,

To close ABRO or just reduce its activities would be a tragedy. It is terrifying to think that the ARC can even contemplate such action, without full consultation with the whole livestock industry. Quite frankly, it makes no sense whatsoever.^58^

This was the quotation that Gavin Strang picked up in his defence of ABRO in the House of Commons, mentioned in the introduction.

Strang's Parliamentary defence of the organization was backed by a few more Scottish MPs, and the MP for North Somerset, where Long Ashton is situated. The ARC was the key target of criticism, blamed for its short-sightedness and lack of transparency in decision making. Bill Shelton, the Parliamentary under-secretary of state for education and science, was eager to place the responsibility on the ARC, and to wash the government's hands of the planned cuts: ‘I assure the House that, to date and no doubt for the future, although I cannot give the figures, the ARC has been treated well financially, as I think it will acknowledge’. Shelton stressed that no decisions had yet been made, and suggested that it was the council's choice to make savings and invest in new programmes, including genetic engineering:

If this decision goes through, I again emphasise that no decision has yet been made, it is proposed to use the present institute field station at Dryden for what are described as 10 highly innovative scientists with a supporting staff of about 40 who would conduct the animal genetic experiments … The ARC made it clear in its press release that this is not a matter of cutting to reduce total expenditure but of cutting to increase flexibility and to allow new sums to be spent on new high priority work in its institutes on agricultural food, nutrition and bio-technology.^59^

In response to the pressure from farming bodies, Parliamentarians and the press, the ARC introduced a consultation process and reached out to stakeholders for advice.^60^ In February and March 1982, the ARC produced two documents outlining its rationale.^61^ According to the first, cattle breeding work was too expensive and inflexible, and much could be saved from selling the cattle farms and focusing instead on sheep and pigs. The latter document reiterated a commitment to smaller-scale, university-based research on animal breeding, suggesting that a much leaner ABRO should instead focus on animal genetics from an applied angle, but with sufficient capacity to include basic science where appropriate. It was, however, produced after the ARC meeting on 23 March, where the council discussed the strong opposition to the cuts and tried to alleviate them somewhat. The envisioned changes would now mean the loss of 139 jobs – sixty scientists, technicians and farm assistants, and seventy-nine administrators and farm workers not directly involved in experiments. Some posts would be freed through early retirement, but most would be lost through redundancy. While it was the council's goal to refocus on basic research, the Hereford Breeding Society weighed in with a defence of the long-term Hereford project. Furthermore, as a customer under the Rothschild arrangement, MAFF representatives objected to curtailing the research that the ministry was now funding.^62^

While recruiting support from various interest groups, King proceeded to plan the future of ABRO. He requested that the heads of departments come up with a plan for maintaining the organization at 85 per cent capacity. The head of reproductive physiology, Gerald Wiener, who had worked at ABRO since 1947, defended his domain. In a memorandum to King, he argued that ‘across-the-board departmental cuts are not the best way of reducing ABRO's total commitments and that in fact the emphasis on research in physiological/biochemical genetics should be strengthened at the expense of other activities’.^63^ Since reproductive physiology was recognized as an excellent area of research by the visiting group, and was the presumed core of the future ABRO, Wiener ended up as a go-between liaising with the ARC. In his correspondence with Jinks and Riley, he emphasized that he wished the cuts could be avoided, but urged ring-fencing experimental work in physiology and the projects run by young scientists. In a further report to the ARC, he noted two cultures, or ‘philosophies’, that coexisted in ABRO: genetic improvement of breeds, in collaboration with farmers, on the one hand, and fundamental research into animal genetics on the other. He suggested that

the difference between the two philosophies is significant and at the root of much of the conflict both within ABRO and with the Council. It underlies the general emphasis that has been given increasingly over recent years to the more applied experiments in ABRO in direct conflict for resources with ideas for more fundamentally oriented research.^64^

Wiener saw ABRO in a fundamental research role, providing advice and close links with industry, including analysis of commercial breeding programmes with quantitative genetics, but not as a site of applied research as such.

Wiener's suggestions appealed to the ARC. As he recalled, at one of the meetings with a senior ARC representative, he had been offered the position of the new ABRO director – the council had decided to replace King. Wiener had cited his age (he had four years left until retirement) and his reluctance to preside over redundancies of colleagues as the main reasons he had declined and suggested Roger Land instead.^65^ Land had joined ABRO in 1966, and worked extensively on predicting reproductive potential through physiological tests. Land's appeal to the ARC was aided by another consideration – unlike both Wiener and King, he was eager to introduce genetic engineering research at the institute.^66^

In April 1982, the ARC reviewed the proposals for a heavy cut. Responding to pressure from multiple stakeholders, including MAFF, it agreed to reduce ABRO's funding by about half – from £2.2 to £1.2 million – not the original 80 per cent. The council also promised an extra fund of about £400,000 for ‘important new work of high scientific merit’, with genetic modification in mind.^67^ ABRO's limited success in reversing some of the cuts was a result of effective networking by its director and outrage at the announcement of cuts without due warning or deliberation. Furthermore, while its financial arrangements with MAFF had been troublesome, the Rothschild system of dual support had proved a useful resource, as MAFF intervened on the organization's behalf –arguably, the ARC would have had less trouble curtailing ABRO programmes before Rothschild. Beyond recruiting allies, however, the management at ABRO decided to align the organization with ARC's new priorities, specifically genetic engineering. By the end of the year, John King was appointed away from ABRO to lead the ARC's Edinburgh Breeding Liaison Group at the Edinburgh School of Agriculture, and Roger Land presided over the restructuring at ABRO, and over setting up a molecular biology programme.

## Going molecular at ABRO

The issue of genetic modification was discussed by the 1980 visiting group. In fact, the suggestion came from King, in his report to the group:

One of the major problems facing ABRO for the future is to decide at what stage to develop studies of genetic engineering. Up till now all attempts to introduce new DNA into germ lines of mammals had been unsuccessful but a recent report (*New Scientist*, 11 September 1980, p. 763), suggests that this hurdle may have been overcome. The general idea is developing so rapidly that an appropriate animal breeding involvement now seems timely. The most suitable means of achieving this objective would be by appointing appropriately qualified staff at ABRO and then seconding them to work by arrangement in a molecular biology unit.^68^

While molecular biology had its origins in the study of protein rather than DNA, and ABRO scientists had worked with other molecules such as hormones, by 1980 and in this context, ‘molecular biology’ meant working with recombinant DNA.^69^ The ‘recent report’ to which King referred concerned genetically modified mice born at Yale and announced at a conference in West Berlin.^70^ On top of manipulation, isolating and eventually sequencing important genes were promising avenues. The ARC's enthusiasm for the technology was palpable, as it organized a meeting on animal genetic engineering in Cambridge in October 1980, and a scientific conference in early 1981.^71^ Indeed, King cited the *New Scientist* report because the Yale group had not published its results in a journal by that point.^72^ Two years later, in December 1982, images of the giant ‘supermice’ made through the insertion of rat growth hormone genes into the mouse embryo made the cover of *Nature* and were picked up enthusiastically by the US and British press. With these animals, a real promise of genetic modification on the farm was being discussed more seriously than ever.^73^

The visiting group agreed that such genetic work would be ‘timely’, but should start on a ‘modest scale’, with close links to the University of Edinburgh. Yet while King expressed enthusiasm in his report, he had strong reservations about adopting genetic engineering at ABRO, as did some of his colleagues. In a 1981 volume celebrating ARC's fiftieth anniversary, King concluded his piece on achievements in animal breeding by commenting that his ‘own opinion would be less optimistic than many that have been advanced … While genetic engineering seems a most legitimate area of enquiry its practical application is likely to lie well into the next 50 years’.^74^ Gerald Wiener, the head of physiological genetics at ABRO, agreed with King's assessment: ‘It is an area we should keep our eyes on, but for the moment, from the outside, in spite of its trendy appeal’.^75^

Roger Land, who took over the directorship, was committed to introducing molecular genetics at the organization. He went on to implement the visiting group's recommendations and in 1983 hired Richard Lathe to start a genetic-engineering programme. A University of Edinburgh graduate, Lathe had moved to France for his graduate studies to work with Pierre Chambon, a prominent French molecular biologist and a European leader in recombinant DNA research. From ABRO's perspective, Lathe could offer rare and valuable experience – he had worked for a biotech company, Transgene SA in Strasbourg. The company had been set up with Chambon's patronage and considerable state funds, and had worked on producing the recombinant vaccine for rabies that was successfully deployed in the wild. With his move to ABRO, Lathe brought new perspectives on what genetic manipulation might achieve.^76^

**Figure 3.**
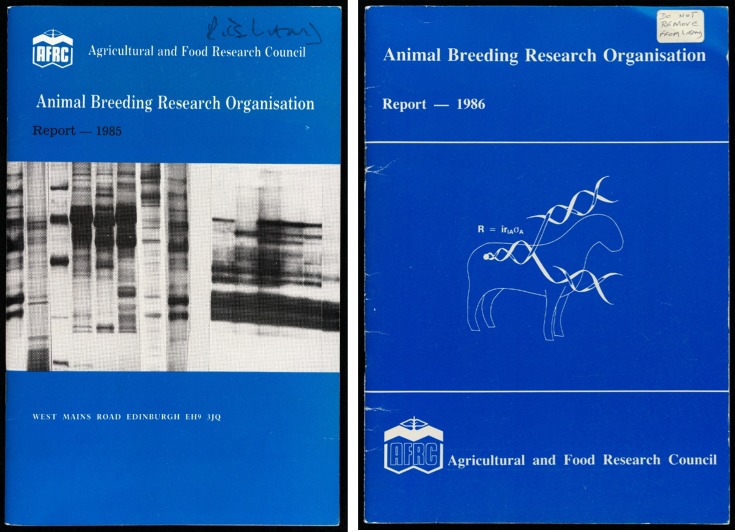
Covers of ABRO annual reports for 1985 (left) and 1986 (right), showing a Southern blot image and a stylized farm animal with replicating DNA respectively. Courtesy of Edinburgh University Main Library, distributed under a CC-BY license.

After the dramatic images of supermice, several groups worldwide looked into extending the techniques to farm animals. The inventors of the mice, the reproductive scientist Ralph Brinster at the University of Pennsylvania and the molecular biologist Richard Palmiter at the University of Washington, initiated a collaboration with the US Department of Agriculture's (USDA) Agricultural Research Service in Beltsville, Maryland, to make transgenic pigs and sheep with growth hormone genes.^77^ Lathe, by contrast, envisioned another strategy, much more in line with what biotech companies had been doing with bacteria: synthesizing valuable medical proteins. While the powerhouse of molecular biology and recombinant DNA research, *E. coli*, showed dramatic successes in producing insulin, human somatostatin and human growth hormone, the majority of animal proteins were difficult to synthesize in bacteria. This is where cattle and sheep came in. In a piece in ABRO's 1984–1985 annual report, Lathe wrote of the ‘molecular tailoring’ of animals.^78^ The cover of the report proudly carried a Southern blot image (a technique invented by Edinburgh's Edwin Southern in 1973 and used to identify isolated genes) rather than the usual photograph of farm animals (Figure 3). Sheep could be modified with genes for useful drugs, with the hope that their expression could be targeted to their milk – an approach that came to be known as ‘pharming’ in the 1990s.^79^

Scientists at leading sites such as the MRC's Laboratory of Molecular Biology in Cambridge were reluctant to adopt a commercial biotech outlook in the early 1980s, and persuading them to do so was a difficult process.^80^ ABRO scientists could not afford such luxury. Significant resources were thrown into the implementation of genetic engineering, and Land's approach was at times heavy-handed. Ian Wilmut, a reproductive physiologist experienced in embryo transfer, was forced to abandon his project on prenatal mortality in sheep and move to the transgenic team.^81^ Alan Archibald, an ABRO scientist who had gone to the European Molecular Biology Laboratory in 1982–1983 to train in recombinant techniques, was in charge of isolating and assembling the gene constructs that were to be injected into sheep and mice.^82^ John Bishop, at the Department of Genetics, was a key senior collaborator, and the molecular biology group also relied on his mouse facilities at the King's Buildings site. Bishop's postdoc John Clarke took over ABRO's molecular biology programme after Lathe left Edinburgh in 1986.

While funding continued to dwindle and decline in the late 1980s, genetic engineering remained a priority. In the preface to AFRC's first corporate plan for 1984–1988 – an exercise in projecting accountability and transparency – Ralph Riley described the council's mission as providing ‘fundamental science from which entirely new technologies will grow in the future’.^83^ He outlined the recent changes to the funding, but also singled out opportunities – specifically that ‘molecular biology, biochemistry and biophysics are providing new understanding and new potentialities for the manipulation and management of crop plants, farm animals and industrial micro-organisms’.^84^ Referring to this trend, Roger Land stated in the 1985 ABRO annual report that ‘genetic manipulation is seen to be ABRO's primary responsibility’.^85^ He also noted that changes in animal breeding were not just about the techniques, but also about the very organization of science – ‘the need to introduce private funding to make up for reduced public expenditure could change the relationship between research and industry’.^86^

Introducing recombinant DNA techniques proved a lifeline for ABRO, although the strategy was not unique to the institution. As discussed above, the Microbiological Research Establishment at Porton Down adopted a similar strategy when faced with the 1970s defence cuts. An appeal to promissory science, in terms of both productive future research and economic outcome, proved effective for both struggling institutions – especially during the early stages of adopting genetic engineering – driven by anxieties about Britain's place in global science and its ability to cultivate innovative research. At the same time, as Jane Maienschein has pointed out, cutting edges cut both ways, and as some research programmes are elevated, other suffer.^87^ Changes at ABRO affected its identity and envisioned beneficiaries; from being an agricultural body attuned to farming needs, the institution reoriented itself towards drug production and molecular research, while its breeding research programmes declined.

## Public–private science

In response to the complex political to-and-fro, ABRO changed its core portfolio and focus, and embraced genetic engineering. In a context of agricultural overproduction, Thatcher's government saw little value in increasing productivity through science, and expected industry to pull more weight in funding research. At this time of uncertainty, the ARC identified plant biotechnology as an area that could supplement a shrinking budget. Meanwhile, ABRO also sought extra funds by entering a relationship with PPL, a private company it helped create. Yet while the changes to the organization's research agendas were largely imposed from London, its entrepreneurial story was specific to Scotland.

After cutting programmes at ABRO and Long Ashton in 1982, the ARC was facing new difficulties. In May 1981, Thatcher's government had announced severe cuts to university budgets. Since the Second Word War, Britain has implemented a ‘dual-support’ system for university research, with separate streams of funding from the research councils and the university ‘block grants’, distributed by the University Grants Committee. The block grant was used for teaching, but also to support research across the sciences and humanities. In the natural sciences, the money was often used to furnish laboratories and to support risky projects before they could receive research council or other funding. Thatcher's government decided to cut the UGC block grant heavily, generating much anxiety about the future of British science.^88^ The Advisory Board for the Research Councils (ABRC), the umbrella body set up to coordinate between all councils and the government after the Rothschild reforms, responded to university cuts with alarm. Giving testimony to the House of Lords Select Committee on Science and Technology, Sir Alec Merrison, ABRC chairman and vice chancellor of the University of Bristol, criticized the UGC cut heavily, suggesting it would undermine efficiency and innovation.^89^

In its 1982 recommendations, the ABRC urged Keith Joseph, the Secretary of State for Education and Science, to maintain (and ideally expand) research council support for university research. Furthermore, the board sought more money for the international commitments of the Science and Engineering Research Council, such as CERN. The ARC, with its minor university funding, saw its interests sacrificed in this process. The ABRC insisted that the council had to make savings of £3 million a year by 1985, about 3.5 per cent of its annual budget. Ralph Riley was a member of the ABRC and, in an unprecedented move, dissented from the recommendations; his response was published as an appendix to the report.^90^

Riley's dissent had little effect; 1982 and 1983 were bad years for the ARC. Five reports to the government, while paying lip service to the customer–contractor principle, strongly criticized the way in which agricultural research was being commissioned. Most placed some of the blame on the ARC.^91^ Furthermore, the council's case was misaligned with government priorities. Since 1973, the UK had been part of the European Common Market, and with it, the Common Agricultural Policy. The late 1970s and the 1980s witnessed a major crisis of overproduction in European agriculture, driving prices down and increasing subsidies. Treasury correspondence regarding the future of the ARC showed little sympathy for supporting agricultural research when it saw extreme production efficiency as part of the problem.^92^ John Moore, economic secretary to the Treasury and self-styled ‘Mr Privatisation’, went as far as suggesting that the ARC should be privatized and act as a major contractor. This option garnered little enthusiasm from the Treasury's civil servants and generated opposition on legal grounds, since forcing the council to go private would contravene its Royal Charter.^93^ The options for privatizing its institutes, on the other hand, were not ruled out, although scepticism over finding buyers was voiced.

The ARC tried to mitigate criticism by following the ABRC's call to fund more university science, and the recommendations from the Advisory Council for Applied Research and Development (ACARD) to support more food research. In 1983, the ARC changed its name to the Agricultural and Food Research Council (AFRC), to reflect the new emphasis. The government expected that the AFRC could find money through more active commercialization of research, and a state-backed Agricultural Genetics Company was set up for the purpose in 1984. It followed the example of the MRC company, Celltech, which commercialized monoclonal antibodies and other technologies developed by the council.^94^ After Thatcher lambasted the failure to patent monoclonal antibodies, the responsible National Research Development Corporation was merged with the National Enterprise Board to form the British Technology Group (BTG).^95^ With Celltech as a model, the BTG funded the new Agricultural Genetics Company and brought in two private players, the oil company Ultramar and Advent, a European venture capital fund set up by Monsanto.^96^ The new Cambridge-based company, however, only dealt with plant genetics, leaving animal research aside.

Meanwhile at ABRO, Rick Lathe, John Bishop and John Clark envisioned the commercialization of the pharming project, in a model increasingly used by US institutions but still novel in Britain. With Roger Land, they planned to patent the key techniques, and sell an exclusive licence to a private company. Since farm animals did not fall into the remit of the Agricultural Genetics Company, ABRO management decided to tap into local developments. London and Cambridge may have taken the lead in British biotechnology, but there was also much activity in Scotland. In particular, the work on the recombinant hepatitis B vaccine by the University of Edinburgh's Ken Murray and Noreen Murray, done in association with the European biotechnology firm Biogen, was paving the way.^97^

In the early 1980s, the Scottish Development Agency (SDA), a new public body set up with North Sea oil money to foster Scottish business, established a biotechnology department. Lathe approached the SDA's Iain Shirlaw, a former food scientist who had retrained at the London Business School. The SDA provided crucial seed funding and attracted venture capital from Prudential Investment Managers, an investment arm of the major insurance company, and from the Transatlantic Capital Bio-sciences Fund. The new company began as Caledonian Transgenics Ltd, and was soon renamed Pharmaceutical Proteins Ltd, or PPL. Technically, it was not a spin-off, as the AFRC institutes were then banned from owning equity in private companies. Instead, PPL was an independent firm that would license ABRO's patents and, crucially, fund some of its research, while also hiring its own scientific staff.^98^

Initially, PPL set up offices and laboratories at the King's Buildings, in close proximity to the molecular biology team of the organization that it now co-funded. Besides receiving investment from PPL, ABRO benefited from letting the new company its facilities, and from charging the firm for instrumentation and overheads, and for use of farm space for the company's experimental sheep. While Roger Land acknowledged concerns about the confidentiality of commercially relevant research – which could no longer be discussed as openly as in the past – he stressed that PPL would provide a ‘substantial sum which will release pressure on core funding and help the Research Station through the period of funding difficulties’. This was, moreover, ‘in line with Government and AFRC policy’.^99^ Grahame Thurnbull, the first CEO of PPL, claimed to appreciate the concerns of the scientists, stating that ‘the absolute need … to be able to publish any future work involving the Company is well understood’.^100^ After PPL hired its own team in 1988, the arrangement moved beyond investment into a long-term collaboration between the AFRC and company scientists, with much movement between the two bodies.

By the time the deal with Caledonian Transgenics/PPL was finalized, ABRO had faced another shake-up as the AFRC was forced to continue its programme of restructuring. In an attempt to save money on administration and to centralize farm animal research, ABRO was united with the eminent Institute of Animal Physiology in Babraham near Cambridge. In addition, it absorbed the AFRC's Poultry Research Centre, based in the village of Roslin just south of Edinburgh. The new, enlarged establishment became the Institute of Animal Physiology and Genetics Research (IAPGR), with the Cambridge and Edinburgh Research Stations, and started operating in 1986. The Edinburgh Research Station was to move all its laboratory operations to Roslin as soon as possible, although the move could only be finalized in 1990.

Despite the cuts and painfully felt redundancies, ABRO and its successors within the IAPGR managed to secure new sources of funding. They tapped into governmental and private enthusiasm about biotechnology with results in the form of the AFRC block grants, MAFF commissions and PPL's investment. The transgenic programme in Edinburgh continued to rely on a research-institute model with its long-term grants that promised a continuity unobtainable in most university laboratories. Since the first cuts to ABRO, molecular biology research was funded through the special extra grant for new science that was part of the 1982 settlement with the ARC. In 1988, the AFRC introduced further special grants to support transgenic and stem cell research, in a funding scheme known as the Transgenic Animal Programme that was available almost exclusively to the Edinburgh and Cambridge stations of the IAPGR.^101^

This diversity of sponsors created a sustained and hybrid alliance between public and private funding for animal biotechnology, which Edinburgh geneticists used to their advantage as much as they could. The transition from farm animal genetics to molecular genetics not only changed the orientation of the institute, but also allowed it to weather further policy changes, such as the 1988 volte-face on applied science that determined that any work deemed too ‘near market’ should be carried out privately and not in state-funded bodies.^102^ By contrast, many other AFRC institutes faced closure, mergers and privatization – most notably the Plant Breeding Institute, bought by Unilever in 1987.^103^ In 1981, ARC supported twenty-two institutes and research stations; by 1991, this number had come down to seven.^104^

## Conclusion

In 1990, a sheep called Tracy was born at the IAPGR's Roslin site. She was the first transgenic sheep to produce significant – indeed, dramatic – amounts of a human protein in her milk. This protein was alpha-1-antiripsin, a drug for certain kinds of emphysema and cystic fibrosis. While she was born at the IAPGR, she also represented a promise for PPL to market the drug in the future. Shortly after the news of her birth, in 1993, the IAPGR was disassembled, with the Babraham Institute regaining its name, and the Edinburgh Research Station becoming the Roslin Institute. In the same year, the AFRC was merged with the biological arm of the Science and Engineering Research Council to form the BBSRC, the Biotechnology and Biological Sciences Research Council. The name change was representative of the general decline in agricultural interests as justification for basic research with strategic goals, and defined a new priority for the application of research – commercial biotechnology.

Tracy embodied a triumphant success of genetic engineering in Scotland that had seemed unlikely a decade earlier. When she died in 1996, her body was stuffed and acquired by the Science Museum in London. Shortly afterwards, Dolly the cloned sheep was announced to the world. Dolly was not genetically modified, but rather a prototype for improving the woefully inefficient production of transgenic animals by cloning sheep from successfully modified cells.^105^ In *Dolly Mixtures*, Sarah Franklin has highlighted ‘the extent to which [Dolly] results from a mixture of agricultural, commercial, industrial and medical purposes’.^106^ While these interactions have a long history, their nexus took place in the 1980s, with the decline of agricultural science funding and a growing emphasis on genetic modification. ABRO was one of the earliest sites, globally, where these connections became fixed and productive, in response to sustained crisis.

It was the promise of biotechnology to the British economy that both devastated the old ABRO and allowed the station to survive in a new guise. While there had been reasons to expect ABRO's eventual move into animal biotechnology, this shift did not result from a coherent policy, but from a set of complex and contingent negotiations that played out on the ground. Furthermore, in seeking new support in the form of PPL investment, ABRO managed to have the best of both worlds for its molecular genetics programme. Private funding not only brought extra income, but also demonstrated the commercial relevance of ABRO research; at the same time, continuous AFRC and MAFF funding offered long-term security unavailable through shorter grant cycles, enabling the organization and its successors to take more risks. This hybrid funding arrangement did come at a cost, however, as multiple sponsors often advanced conflicting agendas, and the Roslin Institute had to spend much energy balancing those in the early 1990s.

In terms of the history of British science and its funding, the case of ABRO highlights both continuities and changes in the 1980s. Developed in line with Thatcherite ideals, privatization and the search for commercial funding were novel features that have left a deep imprint in the life sciences. At the same time, this story cannot be reduced to the diverse and often conflicting policies of Thatcher's governments. Distributed actors at various levels, from research councils to journalists, shaped the outcomes. Many driving factors originated in the 1970s policy decisions, from the Rothschild reforms to the enthusiasm about biotechnology that was sustained in the 1980s. The strategy of embracing novel technology as a way out of a funding crisis pre-dated the 1980s cuts. These cuts were themselves not always a clear policy, but sometimes stemmed from changing accounting mechanisms. Finally, local factors made a difference: ABRO's resistance, its newly entrepreneurial outlook, its ability to benefit from Scottish developments, and internal tensions present within the organization.

## Footnotes

1 Renamed the Agricultural and Food Research Council (AFRC) in 1983 to highlight the importance of food research to the British economy. In 1994, the BBSRC was founded, incorporating the AFRC with the biological programmes of the former Science and Engineering Research Council.

2 This paper builds most directly on Miguel García-Sancho, ‘Animal breeding in the age of biotechnology’, History and Philosophy of the Life Sciences (2015) 37, pp. 282–3042620520110.1007/s40656-015-0078-6. On Dolly see especially Sarah Franklin, Dolly Mixtures: The Remaking of Genealogy, Durham, NC: Duke University Press, 2007. See also Gina Kolata, Clone: The Road to Dolly and the Path Ahead, London: Penguin, 1998; Ian Wilmut, Keith Campbell and Colin Tudge, The Second Creation: Dolly and the Age of Biological Control, Cambridge, MA: Harvard University Press, 2001; Tom Wilkie and Elizabeth Graham, ‘Power without responsibility: media portrayals of British science’, in Arlene Judith Klotzko (ed.), The Cloning Sourcebook, Oxford: Oxford University Press, 2001, pp. 135–150; Richard Holliman, ‘Media coverage of cloning: a study of media content, production and reception’, Public Understanding of Science (2004) 13, pp. 107–130; Dmitriy Myelnikov and Miguel García-Sancho (eds.), Dolly at Roslin: A Collective Memory Event, Edinburgh: The University of Edinburgh, 2017, available online at www.research.ed.ac.uk/portal/files/36020152/CME_web_090517.pdf, accessed 14 July 2017.

3 On science funding in the Thatcher years see Martin Ince, *The Politics of British Science*, Brighton: Wheatsheaf, 1986; Tom Wilkie, British Science and Politics since 1945, Oxford: Blackwell, 1991; Colin Thirtle, Paolo Palladino and Jenifer Piesse, ‘On the organisation of agricultural research in Great Britain, 1945–1994’, Research Policy (1997) 26, pp. 557–576; Paolo Palladino, Plants, Patients, and the Historian: (Re)membering in the Age of Genetic Engineering, New Brunswick, NJ: Rutgers University Press, 2003.

4 Palladino, op. cit. (3); Jean-Paul Gaudillière, ‘The farm and the clinic: An inquiry into the making of our biotechnological modernity’, Studies in History and Philosophy of Biological and Biomedical Sciences (2007) 38, pp. 521–5291754384410.1016/j.shpsc.2007.03.012; see also papers in *Between the Farm and the Clinic: Agriculture and Reproductive Technology in the Twentieth Century*, special issue of Studies in History and Philosophy of Biological and Biomedical Sciences (2007) 38(2)10.1016/j.shpsc.2007.03.00317543834.

5 On Thatcher and science see Jon Agar, ‘Thatcher, scientist’, *Notes and Records of the Royal Society* (2011), at http://rsnr.royalsocietypublishing.org/content/65/3/215, accessed 15 October 2016. On Thatcher and Thatcherism see e.g. Richard Vinen, Thatcher's Britain: The Politics and Social Upheaval of the Thatcher Era, London: Simon & Schuster, 2009; Florence Sutcliffe-Braithwaite, ‘Neo-liberalism and morality in the making of Thatcherite social policy’, Historical Journal (2012) 55, pp. 497–520; Andrew Gamble, ‘The Thatcher myth’, British Politics (2015) 10, pp. 3–15.

6 Martin Kenney, Biotechnology: The University–Industrial Complex, New Haven, CT and London: Yale University Press, 1986; Sally Smith Hughes, ‘Making dollars out of DNA: the first major patent in biotechnology and the commercialization of molecular biology, 1974–1980’, Isis (2001) 92, pp. 541–5751181089410.1086/385281; Hughes, Genentech: The Beginnings of Biotech, Chicago: The University of Chicago Press, 2011. Arnold Thackray (ed.), Private Science: Biotechnology and the Rise of the Molecular Sciences, Philadelphia: University of Pennsylvania Press, 1998, a collection of papers on the history of biotechnology, is also indicative here, even though some papers highlight the role of the state. For a study of British biotechnology told from an entrepreneurial perspective see also Lara V. Marks, The Lock and Key of Medicine: Monoclonal Antibodies and the Transformation of Healthcare, New Haven, CT and London: Yale University Press, 2015.

7 Susan Wright, Molecular Politics: Developing American and British Regulatory Policy for Genetic Engineering, 1972–1982, Chicago and London: The University of Chicago Press, 1994; David C. Mowery, Richard R. Nelson, Bhaven N. Sampat and Arvids A. Ziedonis, Ivory Tower and Industrial Innovation: University–Industry Technology Transfer before and after the Bayh-Dole Act in the United States, Stanford, CA: Stanford Business Books, 2004; Doogab Yi, ‘Who owns what? Private ownership and the public interest in recombinant DNA technology in the 1970s’, Isis (2011) 102, pp. 446–4742207377010.1086/661619.

8 Robert Bud, The Uses of Life: A History of Biotechnology, Cambridge: Cambridge University Press, 1993; Bud, ‘From applied microbiology to biotechnology: science, medicine and industrial renewal’, Notes and Records of the Royal Society (2010) 64, pp. S17–S292097334510.1098/rsnr.2010.0044; Herbert Gottweis, Governing Molecules: The Discursive Politics of Genetic Engineering in Europe and the United States, Cambridge, MA and London: MIT Press, 1998; Soraya de Chadarevian, ‘The making of an entrepreneurial science: biotechnology in Britain, 1975–1995’, Isis (2011) 102, pp. 601–6332244854010.1086/663596; Geoffrey Owen and Michael M. Hopkins, Science, the State, and the City: Britain's Struggle to Succeed in Biotechnology, Oxford: Oxford University Press, 2016.

9 Timothy DeJager, ‘Pure science and practical interests: the origins of the Agricultural Research Council, 1930–1937’, Minerva (1993) 31, pp. 129–150.

10 DeJager, op. cit. (9); Thirtle, Palladino and Piesse, op. cit. (3).

11 The National Agricultural Advisory service became the Agricultural Development Advisory Service, or ADAS, in 1971.

12 Forbes W. Robertson, ‘Genetics’, Proceedings of the Royal Society of Edinburgh, Section B, Biological Sciences (1983) 84, pp. 211–229; Clare Button, ‘James Cossar Ewart and the origins of the Animal Breeding Research Department in Edinburgh, 1895–1920’, *Journal for the History of Biology* (forthcoming).

13 John W.B. King, ‘Animal breeding research in Britain, 1931–1981’, in G.W. Cooke (ed.), Agricultural Research, 1931–1981: A History of the Agricultural Research Council and a Review of Developments in Agricultural Science during the Last Fifty Years, London: Agricultural Research Council, 1981, pp. 277–288; H.P. Donald, Foreword, in ‘Animal Breeding Research Organisation Report – 1963’, EUA IN23/1/1/2, Records of Roslin Institute and predecessor institutions, Centre for Research Collections, Main Library, University of Edinburgh, Edinburgh, UK (‘Roslin papers’). The multibreed project is discussed in St Clair S. Taylor, ‘A multibreed approach to breed comparison’, ‘Animal Breeding Research Organisation Report – January 1972’, EUA IN23/1/1/2, Roslin papers.

14 Margaret M. Dunlop, Goodbye Berlin: The Biography of Gerald Wiener, Edinburgh: Birlinn, 2016, p. 162.

15 Miles Parker, ‘The Rothschild report (1971) and the purpose of government-funded R & D: a personal account’, Palgrave Communications (2016) 2, article number 16053, at www.nature.com/articles/palcomms201653, accessed 15 October 2016; anon., ‘Another fresh look’, Nature (1970), 228, p. 1244.

16 On Heath's government machinery reform see Kevin Theakston, ‘The Heath government, Whitehall and the civil service’, in Stuart Ball and Anthony Seldon (eds.), The Heath Government, 1970–1974: A Reappraisal, London and New York: Longman, 1996, pp. 75–106; John Ramsden, ‘The prime minister and the making of policy’, in ibid., pp. 21–46. On Rothschild see Kenneth Rose, Elusive Rothschild: The Life of Victor, Third Baron, London: Weidenfeld & Nicolson, 2003; Parker, op. cit. (15). On the Think Tank see also Tessa Blackstone and William Plowden, Inside the Think Tank: Advising the Cabinet, 1971–1983, London: Heinemann, 1988.

17 Victor Rothschild, ‘The organisation and management of government R. & D.’, in A Framework for Government Research and Development (Cmnd. 4814), London: Her Majesty's Stationery Office, 1971.

18 ‘Two views of British science’, Nature (1971) 234, p. 169. On the Rothschild report and its implications see especially Neil Calver and Miles Parker, ‘The logic of scientific unity? Medawar, the Royal Society and the Rothschild controversy 1971–72’, Notes and Records of the Royal Society (2016) 70, pp. 83–1002701768110.1098/rsnr.2015.0021PMC4759716; and Parker, op. cit. (15). See also Wilkie, op. cit. (3), pp. 80–93; Soraya de Chadarevian, Designs for Life: Molecular Biology after World War II, Cambridge: Cambridge University Press, 2002, pp. 339–62; Agar, op. cit. (5).

19 On pure/applied research and various other definitions see Robert Bud, ‘Framed in the public sphere: tools for the conceptual history of “applied science” – a review paper’, History of Science (2013) 51, pp. 413–433; Sabine Clark, ‘Pure science with a practical aim: the meanings of fundamental research in Britain, circa 1916–1950’, Isis (2010) 101, pp. 285–3112071827310.1086/653094. Rothschild elaborated his definitions and taxonomy of pure/applied research in Victor Rothschild, ‘Pure and applied research: the Trueman Wood lecture to the Royal Society of Arts, 8 December 1971’, in Rothschild, Meditations of a Broomstick, London: Collins, 1977, pp. 68–83; Rothschild, ‘Forty-five varieties of research (and development)’, Nature (1972) 239, pp. 373–3781263529010.1038/239373a0.

20 Rothschild, ‘Pure and applied research’, op. cit. (19), p. 73.

21 Wilkie, op. cit. (3), pp. 87–88.

22 The Department of Agriculture and Fisheries for Scotland and its institutes were spared in the Rothschild report and subsequent reforms in the early 1980s, as its grants-in-aid model was considered efficient and not to be tampered with. Issues of Scottish autonomy also featured.

23 Rothschild, op. cit. (17), p. 21.

24 Agricultural Research Council (ARC), *Planning for Future Flexibility and Structural Change*, London: Agricultural Research Council, 1982, p. 16.

25 H.P. Donald, ‘New tasks for livestock genetics’, in *Animal Breeding Research Organisation Report: January 1974*, pp. 7–11, 8.

26 Quoted in director's report for the 1980 visiting group to ABRO, Box 14, BBSRC archives, Swindon, p. 1.

27 William Henderson, ‘1972–1980: post-Rothschild’, in G.W. Cooke (ed.), Agricultural Research, 1931–1981: A History of the Agricultural Research Council and a Review of Developments in Agricultural Science during the Last Fifty Years, London: Agricultural Research Council, 1981, pp. 277–288; Thirtle, Palladino and Piesse, op. cit. (3); Parker, op. cit. (15).

28 Joint Consultative Organisation for Research and Development in Agriculture and Food (JCO), Second Reports of the Joint Consultative Organisation for Research and Development in Agriculture and Food, London: Her Majesty's Stationery Office, 1975, paragraph 61(iii).

29 Not to be confused with the Priorities Board that replaced the Joint Consultative Organisation in 1984.

30 JCO, Reports of the Joint Consultative Organisation for Research and Development in Agriculture and Food 1976–77, London: Her Majesty's Stationery Office, 1977.

31 Richard B. Flavell, ‘Obituary: Sir Ralph Riley. 23 October 1924–27 August 1999’, Biographical Memoirs of Fellows of the Royal Society (2003) 49, pp. 385–396. The chairmen of the ARC tended to be aristocrats with an interest in farming: Lord Porchester (later the Earl of Carnarvon), 1978–1982, and then the Earl of Selbourne in 1982–1989.

32 Henderson, op. cit. (27); Parker, op. cit. (15).

33 Lord Rothschild to Margaret Thatcher, 20 June 1983, the National Archives of the UK (TNA): T 494/80 Treasury: Agricultural Research Council, review of agricultural and organisational policy.

34 Thatcher to Rothschild, 29 July 1983, TNA: T 494/80 Treasury: Agricultural Research Council, review of agricultural and organisational policy.

35 See Agar, op. cit. (5). In her memoirs Thatcher claimed, ‘As someone with a scientific background, I knew that the greatest economic benefits of scientific research had always resulted from advances in fundamental knowledge rather than the search for specific applications’. Margaret Thatcher, The Autobiography, London: Harper Press, 1993, p. 592.

36 ARC, Report of the Agricultural Research Council for the Year 1978/79, London: Her Majesty's Stationery Office, 1979, p. 1.

37 ARC, Report of the Agricultural Research Council for the Year 1979/80, London: Her Majesty's Stationery Office, 1980, p. 1.

38 ARC, Report of the Agricultural Research Council for the Year 1980/81, London: Her Majesty's Stationery Office, 1981, p. 1.

39 Robert Walgate, ‘UK research councils: getting off lightly’, Nature (1981) 289, pp. 435–436.

40 On cash planning see Colin Thain and Maurice Wright, ‘The advent of cash planning’, Financial Accountability & Management (1989) 5, pp. 149–162; Thain and Wright, The Treasury and Whitehall: The Planning and Control of Public Expenditure, 1976–1993, Oxford: Clarendon Press, 1995, pp. 352–403; Andrew J. McLaughlin and Jeremy J. Richardson, ‘Learning to live with public expenditure: politics and budgeting in Britain since 1976’, Public Budgeting and Financial Management (1994) 6, pp. 97–129.

41 ARC, Report of the Agricultural Research Council for the Year 1981/82, London: Her Majesty's Stationery Office, 1982, p. 2.

42 On continuity and change in the new biotechnology see Bud, *The Uses of Life*, op. cit. (8); Jean-Paul Gaudillière, ‘New wine in old bottles? The biotechnology problem in the history of molecular biology’, Studies in History and Philosophy of Biological and Biomedical Sciences (2009) 40, pp. 20–281926887110.1016/j.shpsc.2008.12.004.

43 On US biotechnology see Kenney, op. cit. (6); Eric J. Vettel, Biotech: The Countercultural Origins of an Industry, Philadelphia: University of Pennsylvania Press, 2006; Hughes, *Genentech*, op. cit. (6); Nicolas Rasmussen, Gene Jockeys: Life Science and the Rise of Biotech Enterprise, Baltimore: Johns Hopkins University Press, 2014; Doogab Yi, The Recombinant University: Genetic Engineering and the Emergence of Stanford Biotechnology, Chicago and London: The University of Chicago Press, 2015. On recombinant DNA debates and resulting regulation policy in the US and the UK see Sheldon Krimsky, Genetic Alchemy: A Social History of the Recombinant DNA Controversy, Cambridge, MA: MIT Press, 1984; Wright, op. cit. (7); Gottweis, op. cit. (8).

44 Alfred Spinks, Biotechnology: Report of a Joint Working Party, London: Her Majesty's Stationery Office, 1980.

45 Bud, ‘From applied microbiology to biotechnology’, op. cit. (8). See also Brian Balmer and Margaret Sharp, ‘The battle for biotechnology: scientific and technological paradigms and the management of biotechnology in Britain in the 1980s’, Research Policy (1993) 22, pp. 463–478; Gottweis, op. cit. (8); de Chadarevian, op. cit. (8); Owen and Hopkins, op. cit. (8).

46 ARC, op. cit. (24); see also the testimony of Lord Selbourne, the ARC's chairman, 1982–1989, to the House of Lords: House of Lords Select Committee on Science and Technology, *Science and Government*, vol. 2: *Evidence*, London: Her Majesty's Stationery Office, 1981, p. 84.

47 ARC, op. cit. (24); ARC, op. cit. (41), pp. 1–3.

48 ARC, op. cit. (38), p. 86. Counted by ARC's current expenditure at the time, excluding capital expenditure and the institutions’ receipts from other sources.

49 Animal Breeding Research Organisation: report of the visiting group, 1980, ARC 226/80, Box 14, BBSRC Archives (BBSRC archives), Swindon, p. 9. Please note that the BBSRC has kindly granted access to its archives on the condition of keeping any information regarding specific individuals anonymous. Wherever individuals are identified in this paper, it is from other sources, which include published ARC materials. The surprise of the visiting group members is recorded in ARC, op. cit. (24), p. 45.

50 Jinks is identified as chairman of the 1980 visiting group by Gerald Wiener, interviewed by Clare Button on 16 September 2014, EUA CA15/3, ‘Oral history recordings made as part of the “Towards Dolly” project’, Centre for Research Collections, Main Library, University of Edinburgh (‘“Towards Dolly” oral histories’). Anne McLaren was identified by Ian Wilmut when interviewed by Grahame Bulfield on 22 January 2015, EUA CA15/8, ‘Towards Dolly’ oral histories, and in his comments in Myelnikov and García-Sancho, op. cit. (2).

51 ‘Animal Breeding Research Organisation: report of the visiting group, 1980’, op. cit. (49), p. 17.

52 ‘In confidence: visit of … to Animal Breeding Research Organisation and the Poultry Research Centre, 19th and 20th February 1981’, Box 14, BBSRC archives, p. 1.

53 ‘In confidence’, op. cit. (52), p. 1.

54 Jon Agar and Brian Balmer, ‘Defence research and genetic engineering: fears and dissociation in the 1970s’, in Don Leggett and Charlotte Sleigh (eds.), Scientific Governance in Britain, 1914–79, Manchester: Manchester University Press, 2016, pp. 122–143.

55 Agar and Balmer, op. cit. (54), p. 44. King's proactive role is confirmed by Gerald Wiener and Alan Archibald, then ABRO scientist and the representative for the Institution of Professional Civil Servants, the scientists’ trade union. Wiener interview, op. cit. (50); Alan Archibald interviewed by Grahame Bulfield, 10 November 2014, EUA CA15/5, ‘Towards Dolly’ Oral Histories.

56 Judy Redfearn, ‘Endangered duo’, Nature (1981) 294, p. 685; anon., ‘Agriculture researchers face the dole’, *New Scientist* (24–31 December 1981) 92(1285–1286), p. 851.

57 The bodies whose representatives offered comments in defense of ABRO were the National Cattle Breeders’ Association, the National Sheep Association, the National Pig Breeders’ Association, the Pig Improvement Company, the Meat and Livestock Commission, the Milk Marketing Board and the Hereford Herdbook. Harry Hope, ‘Research Council wields its axe’, *Farmers Weekly*, 18 December 1981, pp. 32–33.

58 Hope, op. cit. (57) , p. 32.

59 15 Parl. Deb. H.C. (6th ser.) (1981–82), pp. 694–706, 703.

60 Lord Porchester (chairman of ARC) to Sir Geoffrey Howe (Chancellor of the Exchequer), 23 March 1982, TNA: T 494/80 Agricultural Research Council, review of agricultural and organisational policy. The Treasury civil servants reacted to the letter from Lord Porchester with confusion, and understood that he was reaching out to Howe in his capacity as an MP who had expressed interest in the ARC, not as the Chancellor.

61 ARC, The Future Programmes and Structure of the Animal Breeding Research Organisation and the Long Ashton Research Station, London: Agricultural Research Council, 1982; ARC, op. cit. (24).

62 Judy Redfearn, ‘Agricultural Research Council savings plans’, Nature (1982) 296, p. 484.

63 Wiener to King, 28 January 1982, ‘Gerald Wiener – photocopied documents relating to ABRO reorganisation (1980)’, Coll-1701, ‘Papers of Gerald Wiener’, Centre for Research Collections, Main Library, University of Edinburgh.

64 ‘Report compiled by Gerald Wiener for ARC’, n.d., ‘Gerald Wiener – photocopied documents’, op. cit. (63).

65 Wiener interview, op. cit. (50); Dunlop, op. cit. (14), pp. 164–168.

66 William G. Hill, ‘Obituary: Roger Burton Land’, Royal Society of Edinburgh Yearbook (1989) Session 1987–88, pp. 61–62.

67 ARC, op. cit. (61), paragraph 8.

68 Director's report for the 1980 visiting group to ABRO, Box 14, BBSRC archives, p. 9.

69 On the origins of molecular biology see Pnina G. Abir-Am, ‘Themes, genres and orders of legitimation in the consolidation of new scientific disciplines: deconstructing the historiography of molecular biology’, History of Science (1985) 23, pp. 73–1171161168810.1177/007327538502300103; de Chadarevian, op. cit. (18).

70 Harold M. Schmeck Jr, ‘3 at Yale report key transplants of genes to mice’, *New York Times*, 3 September 1980, p. A1.

71 The October 1980 meeting is mentioned in Wiener's ‘Report compiled for the ARC’, op. cit. (64). The programme of the second Meeting on Genetic Engineering in Domestic Animals, April 1981, is in Conference Literature 1981, Add. 83883, Anne McLaren papers, British Library, London.

72 Stephanie Yanchinski, ‘Opening the Pandora's box of biology’, *New Scientist*, 11 September 1980, p. 763; Jon W. Gordon, George A. Scangos, Diane J. Plotkin, James A. Barbosa and Frank H. Ruddle, ‘Genetic transformation of mouse embryos by microinjection of purified DNA’, Proceedings of the National Academy of Sciences of the United States of America (1980) 77, pp. 7380–7384626125310.1073/pnas.77.12.7380PMC350507.

73 Dmitriy Myelnikov, ‘Transforming mice: technique and communication in the making of transgenic animals, 1974–1988’, PhD thesis, University of Cambridge, 2015, available at www.repository.cam.ac.uk/handle/1810/253309.

74 King, op. cit. (13), p. 288.

75 Wiener, ‘Report compiled for the ARC’, op. cit. (64).

76 John Bishop and Richard Lathe interviewed by the author on 1 March 2016 in Edinburgh.

77 Robert E. Hammer, Vernon G. Pursel, Caird E. Rexroad, Robert J. Wall, Douglas J. Bolt, Karl M. Ebert, Richard D. Palmiter and Ralph L. Brinster, ‘Production of transgenic rabbits, sheep and pigs by microinjection’, Nature (1985) 315(6021), pp. 680–683389230510.1038/315680a0.

78 Rick Lathe, ‘Molecular tailoring of the farm animal germline’, ‘Animal Breeding Research Organisation Report – 1985’, EUA IN23/1/1/2, Roslin papers, pp. 7–10.

79 García-Sancho, op. cit. (2).

80 De Chadarevian, op. cit. (8).

81 Wilmut interview, op. cit. (50); Myelnikov and García-Sancho, op. cit. (2), p. 1. Paul Simons had moved to ABRO from the MRC Mammalian Genomes Unit at the University of Edinburgh. A young researcher, Paul Simons had also been recruited to work with Wilmut and perform the hands-on embryonic manipulation.

82 Archibald interview, op. cit. (55).

83 Agricultural and Food Research Council (AFRC), *Corporate Plan, 1984–88*, London: Agricultural and Food Research Council, 1983, p i.

84 AFRC, op. cit. (83), p. iii.

85 Roger Land, ‘Comment’, ‘Animal Breeding Research Organisation Report – 1985’, EUA IN23/1/1/2, Roslin papers, p. 4.

86 Land, op. cit. (85), p. 5.

87 Jane Maienschein, ‘Cutting edges cut both ways’, Biology and Philosophy (1994) 9, pp. 1–24.

88 Universities were, on average, to lose 15 per cent of the block grant over three years, but a further 2 per cent cut was imposed in 1983. While the UGC cuts targeted universities as institutions – part of Thatcher's campaign to reform higher education and attack professional autonomy – one unintended consequence was a blow to university science. Geoffrey Walford, ‘The privatisation of British higher education’, European Journal of Education (1988) 23, pp. 47–64; Wilkie, op. cit. (3), pp. 99–102.

89 House of Lords Select Committee on Science and Technology, op. cit. (46), pp. 61–72.

90 Given the gravity of the situation, ABRC took the unprecedented step of publishing this document: Advisory Board for the Research Councils (ABRC), The Science Budget: A Forward Look, London: Her Majesty's Stationery Office, 1982. See also Ince, op. cit. (3), pp. 37–60.

91 Cooper-Lybrand, a consulting firm, criticized poor monitoring of outcomes and revived the case for moving all ARC research to MAFF. Advisory Council for Applied Research and Development insisted the ARC change direction to support more food research, and the council changed its name to Agricultural and Food Research Council (AFRC) to reflect the change in 1983. The House of Commons Select Committee on Agriculture, the Joint Consultative Organisation and the ABRC criticized the double ARC/MAFF bureaucracy as duplicating its effort. The government struggled to respond to all the reports in time, and opinion was split over how to proceed.

92 In the Treasury's position note for the financial secretary, the £170–180 million annual expenditure on agricultural research was described as ‘too high at the time of rising agricultural surpluses’. G.M. Binns to Michael Faulkner, ‘Agricultural Research’, 25 November 1983, TNA: T494/139: Agricultural Research Council: Review of agricultural and organisational policy, p. 7. On the Common Agricultural Policy and food surpluses see John Martin, The Development of Modern Agriculture: British Farming since 1931, Basingstoke: Palgrave Macmillan, 2000, pp. 133–166; Charles W. Capstick, ‘British agricultural policy under the CAP’, in Christopher Ritson and David Harvey (eds.), The Common Agricultural Policy and the World Economy, Wallingford: CAB International, 1991, pp. 71–87.

93 G.M. Binns to Bostock, ‘Agricultural Research Council: Future’, 1 November 1983, TNA: T 494/80 Agricultural Research Council: Review of agricultural and organisational policy. The option to privatize ARC institutes was left open, and followed in the late 1980s.

94 Mark Dodgson, *Celltech: The First Ten Years of a Biotechnology Company*, Brighton: Science and Policy Research Unit, University of Sussex, 1990; de Chadarevian, op. cit. (8).

95 Monoclonal antibodies are highly specific antibodies that are made in multiple identical immune cells. They were developed at the LMB in Cambridge by César Milstein and Georges Köhler in 1975. The case for patenting was presented to the National Research Development Corporation (NRDC), which decided against patenting, citing lack of clear applications. In 1979, scientists at the Philadelphia-based Wistar Institute received a US patent on the making of monoclonal antibodies against tumours and viral parts. Thatcher became a vehement critic of NRDC's failure to secure intellectual property. See de Chadarevian, op. cit. (18), pp. 353–362; de Chadarevian, op. cit. (8); Marks, op. cit. (6), esp. pp. 25–46; see also Marks's online exhibition, ‘The story of César Milstein and Monoclonal Antibodies’, at www.whatisbiotechnology.org/exhibitions/milstein, accessed 21 July 2017.

96 Tim Beardsley, ‘Plant biotechnology: UK company's delayed budding’, Nature (1983) 304, p. 296. The Treasury papers regarding the origins of the Agricultural Biotechnology Company are in TNA: T494/137 Agricultural Research Council proposed agricultural genetics company.

97 Farah Huzair and Steve Sturdy, ‘Biotechnology and the transformation of vaccine innovation: the case of the hepatitis B vaccines, 1968–2000’, Studies in History and Philosophy of Biological and Biomedical Sciences (2017) 64, pp. 11–212851106810.1016/j.shpsc.2017.05.004PMC5541201.

98 Iain Shirlaw, personal communication; Bishop and Lathe interview, op. cit. (76); Peter Newmark, ‘Protein production in transgenic animals’, Nature Biotechnology (1987) 5, p. 874.

99 Roger Land, memorandum, 11 March 1987, ‘File relating to collaboration with PPL on sheep work, 1987–1990’, EUA IN23/3/4/1/4, Roslin papers.

100 Graham Turnbull to John Clark, November 1988, ‘File relating to collaboration with PPL on sheep work, 1987–1990’, EUA IN23/3/4/1/4, Roslin papers.

101 ‘File re: TAP grants, 1988–91’, EUA IN23/3/3/1/2, Roslin papers.

102 Nicholas Read, ‘The “near market” concept applied to UK agricultural research’, Science and Public Policy (1989) 16, pp. 233–238; Wilkie, op. cit. (3), pp. 98–99.

103 Andrew Webster, ‘Privatisation of public sector research: the case of a plant breeding institute’, Science and Public Policy (1989) 16, pp. 224–232; Palladino, op. cit. (3), pp. 64–8.

104 ARC, op. cit. (38); Tom Blundell, ‘Agricultural research: 60 years of achievement’, Science in Parliament (1991) 48, pp. 23–28. In addition, in 1981 the ARC supported four university units and the Soil Survey of England and Wales, and made grants to the London Zoo, Wye College Department of Hop Research, the Commonwealth Agricultural Bureaux and the Edinburgh Regional Computing Centre.

105 García-Sancho, op. cit. (2).

106 Franklin, op. cit. (2), p. 43.

